# Consumer-grade UAV imagery facilitates semantic segmentation of species-rich savanna tree layers

**DOI:** 10.1038/s41598-023-40989-7

**Published:** 2023-08-24

**Authors:** Manuel R. Popp, Jesse M. Kalwij

**Affiliations:** 1 Institute of Geography and Geoecology, Karlsruhe Institute of Technology, Reinhard-Baumeister-Platz 1, 76131 Karlsruhe, Germany; 2 Centre for Ecological Genomics & Wildlife Conservation, Department of Zoology, University of Johannesburg, Auckland Park, Johannesburg, South Africa; 3 Van Hall Larenstein University of Applied Sciences, Velp, The Netherlands

**Keywords:** Ecological modelling, Tropical ecology, Image processing, Machine learning

## Abstract

Conventional forest inventories are labour-intensive. This limits the spatial extent and temporal frequency at which woody vegetation is usually monitored. Remote sensing provides cost-effective solutions that enable extensive spatial coverage and high sampling frequency. Recent studies indicate that convolutional neural networks (CNNs) can classify woody forests, plantations, and urban vegetation at the species level using consumer-grade unmanned aerial vehicle (UAV) imagery. However, whether such an approach is feasible in species-rich savanna ecosystems remains unclear. Here, we tested whether small data sets of high-resolution RGB orthomosaics suffice to train U-Net, FC-DenseNet, and DeepLabv3 + in semantic segmentation of savanna tree species. We trained these models on an 18-ha training area and explored whether models could be transferred across space and time. These models could recognise trees in adjacent (mean F1-Score = 0.68) and distant areas (mean F1-Score = 0.61) alike. Over time, a change in plant morphology resulted in a decrease of model accuracy. Our results show that CNN-based tree mapping using consumer-grade UAV imagery is possible in savanna ecosystems. Still, larger and more heterogeneous data sets can further improve model robustness to capture variation in plant morphology across time and space.

## Introduction

A response of woody vegetation to a change in ecological drivers is often only visible over multiple years^1,2^. Monitoring such trends through repeated sampling is required to capture short-term effects and potential long-term trends following a change in environmental factors. Additionally, the covered area must be sufficiently large to provide environmental context and to cancel out smaller-scale noise in the data^3,4^. Conventional forest inventory methods, however, are limited regarding these aspects due to high time and work intensity.

Remote sensing provides a cost-effective solution enabling extensive spatial coverage with high sampling frequency over prolonged periods^5,6^. In recent years, artificial neural networks, such as convolutional neural networks (CNNs), have been increasingly used^7^. CNNs are a class of artificial neural networks particularly suitable to analyse images. Combined with high-resolution remote sensing data, these networks can enable automated vegetation monitoring at a species level^8–10^. Aeroplanes or unmanned aerial vehicles (UAV) sensors can obtain high-resolution imagery. A UAV usually offers higher operational flexibility and low ground sampling distance at a relatively low cost^11–13^. Consumer-grade UAV imagery has been successfully used to train CNNs for mapping species-specific tree canopy cover in forest ecosystems with high accuracy^14–16^. U-Net is one of multiple CNN architectures frequently used for this purpose^10,17^. A more resource-intense alternative is FC-DenseNet, which uses blocks with dense connections between layers, producing even more accurate predictions of tree species^18,19^. Recent studies employed variations of the DeepLabv3 + architecture^10,19^. This architecture uses atrous spatial pyramid pooling to integrate context on a variable scale without compromising computational efficiency^20^. The successful application of these CNN architectures indicates a potential for long-term monitoring of species composition^9,10^.

Semantic segmentation using CNNs works well for data sets containing few classes, separable through distinct features^7,10^. Therefore, trees with a characteristic crown shape and structure, e.g., coniferous trees, are relatively easy to classify^10^. Moreover, class frequencies of CNN training data are ideally balanced, facilitating accurate prediction of all classes^21^. Best results are thus achieved for agricultural fields, urban areas, and managed forests with few species and consistent features within classes^16,19,22,23^. Savanna ecosystems, however, host considerable taxonomical and morphological diversity^24,25^.

Savanna ecosystems are landscapes comprising of grassland with scattered trees, occupy one-fifth of the Earth’s land surface, and provide important habitat for wildlife and livestock^26,27^. Savannas are also among the ecosystems most sensitive to future land-use changes and climate^26^. Important drivers of savanna ecosystem dynamics comprise large herbivores, fire regimes, and management practices^27,28^. The interplay of such factors makes savannas highly dynamic ecosystems, especially in Southern Africa^28^. Savanna ecosystem functioning and, hence, providing ecosystem services greatly depends on the vitality and composition of its vegetation layer^27,29^. Therefore, efficient methods to monitor trends in savanna vegetation dynamics are required. However, to what extent the replacement of forest inventories by a fully automated approach is feasible for savanna ecosystems remains unclear.

Savanna vegetation is heterogeneous regarding tree species composition, age, height, and spatial arrangement, with tree species showing high phenotypic plasticity^30,31^. Savanna trees are regularly affected by browsing and fire, causing diverse morphology of adult plants due to damage and stress-induced altered growth behaviour^32,33^. This combination of heterogeneous communities, a large number of species, and irregular tree crown shapes makes species-level semantic segmentation complex and challenging^10^.

The introduction of an automated approach to monitoring vegetation dynamics is only viable if it saves effort—All things considered—Compared to a conventional method. A general limitation of deep learning is the need for extensive training data sets^34^. This reduces the benefits of automated species identification if an ad hoc trained CNN is required since manual delineation of tree classes for training and evaluating such models is time intensive. While data sets for species-poor or managed forest stands can be labelled using aerial images alone^35^, species-rich or poorly studied ecosystems may require labour-intensive field campaigns to collect ground truth information. Thus, it needs to be tested whether an automated approach to tree species mapping produces an output of sufficiently high quality with a small training dataset.

A suitable area to test the performance of CNNs on savanna vegetation comprises typical species in a natural or extensively managed habitat where browsers and fire affect woody vegetation. Such areas can be found in the Waterberg region (Northern Sotho: *Thaba Meetse*) in northern South Africa^36–38^. The Waterberg represents a considerable area of the savanna biome of southern Africa and is dominated by veld types characteristic of mountainous savanna. Here, extensively managed nature reserves are located, providing suitable areas for training and quality assessment of CNNs with savanna vegetation. A research project in Lapalala Wilderness—aimed at experimentally testing the impact of elephant reintroduction on savanna dynamics—provided an opportunity to test such methods under controlled field conditions.

In this study, we tested CNN-based semantic segmentation of savanna tree species on orthomosaics generated from UAV imagery with regards to (1) spatial transferability of models within an ecoregion, (2) training and use of a model within a spatially restricted area, and (3) transferability of the second model over time. Additionally, we tested whether class-specific F1-scores were affected by factors concerning the quantity and distribution of the covered area, using metrics mean patch area, total class area, and compactness of the patches.

## Results

### Computing effort

The CNN architectures varied considerably in computing effort and time. Model training with tiles of 512 $\times$ 512 pixels ran at 180 s per epoch for DeepLabv3 + . In contrast, FC-DenseNet training took the longest, with 927 s per epoch (Table 1). Consequently, training each FC-DenseNet took approximately 78 h, split into multiple runs of 24 h for technical reasons. U-Net and DeepLabv3 + were trained in single runs of less than 24 h. Model inference took about 0.7–1.3 h ha^−1^. Due to limited memory, GPU could not be used for the largest tile size during model inference, increasing computing time by one order of magnitude.

**Table 1:** Number of training epochs and mean training time in seconds per epoch with standard deviation (SD).

Architecture	Tile size	BS	Epochs	Seconds per epoch ± SD
U-Net	256	256	299	196.0 ± 1.9
	512	16	161	198.7 ± 1.9
	1024	4	299	199.7 ± 4.5
FC-DenseNet	256	16	256	940.3 ± 4.5
	512	4	295	927.3 ± 6.5
	1024	1	236	914.2 ± 2.3
DeepLabv3 +	256	256	299	197.5 ± 1.7
	512	16	299	179.8 ± 1.5
	1024	4	299	176.1 ± 1.5

Tile size is both number of rows and number of columns of the input image matrix of the respective model. BS is the batch size at which the model was trained. For FC-DenseNet, smaller batch sizes were chosen due to the models high GPU memory requirements. The number of training epochs was restricted to a maximum of 300. Final models were selected by maximum mean intersection over union on the validation data. Mean training time per epoch was calculated for epochs 2–150.

### Model performance

**Figure 1:**
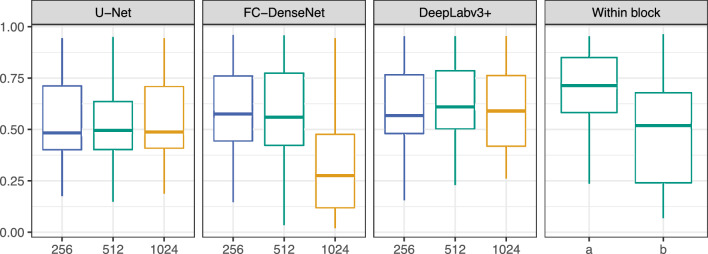
F1-Scores of the models on specific test data sets. The three panels on the left compare the performance of U-Net, FC-DenseNet and DeepLabv3 + trained on orthomosaics of Blocks 1, 2, and 3 with different input tile dimensions (256, 512, and 1024 pixel width and height) and tested on plots 4, 6, and 7. The rightmost panel shows F1-scores of DeepLabv3 + trained with 512 × 512 pixel tiles from 15 ha of Blocks 1, 2 and 3 recorded in 2021 (a) tested on the remaining 3 ha of the same blocks, and (b) tested on the 2022 images of these 3 ha. Colours indicate input tile dimensions.

Model performance on the test data set varied strongly between classes (Table 2). The background class, comprising bare soil, rocks, and herbs, was predicted with very high accuracy by all models. In contrast, F1-Scores for species such as *Commiphora mollis* and *Terminalia sericea*, as well as for class “Other woody vegetation”, were low. Input tile dimensions did not have a consistent effect on the overall performance of the models (Fig. 1, Supplementary Table E1). For U-Net and DeepLabv3 + , there was no significant effect of tile dimensions on F1-Scores. FC-DenseNet with 1024 × 1024 pixel tiles produced the poorest result, with a mean F1-Score of 0.28 and a maximum of 0.94 for the background class. For smaller tile dimensions, FC-DenseNet F1-Scores were similar to that of the other CNNs.

**Table 2:** F1-Scores per class, their mean, as well as overall accuracy for predictions on the three 1-ha test plots of CNN architectures trained with square tiles of 256, 512 and 1024 pixel side length.

F1-Score	U-Net			FC-DenseNet			DeepLabv3 +			(a)	(b)
	256	512	1024	256	512	1024	256	512	1024		
Other	0.31	0.31	0.29	0.38	0.38	0.18	0.41	**0.44**	0.39	0.24	0.16
*Burkea africana*	0.80	0.80	0.75	0.81	0.84	0.21	0.82	**0.85**	0.81	0.87	0.81
*Combretum apiculatum*	0.78	0.76	0.76	**0.83**	**0.83**	0.49	0.82	0.82	0.82	0.82	0.53
*Combretum molle*	0.61	0.62	0.58	**0.70**	0.67	0.47	0.67	0.69	**0.70**	0.67	0.50
*Combretum zeyheri*	0.47	0.43	0.44	**0.56**	**0.56**	0.10	0.50	0.50	0.44	0.45	0.12
*Commiphora mollis*	0.18	0.15	0.19	**0.33**	0.31	0.03	0.16	0.32	0.27	0.50	0.15
*Dichrostachys cinerea*	0.47	0.47	0.41	0.61	0.63	0.49	0.65	0.60	**0.64**	0.84	0.65
*Diplorhynchus condylocarpon*	0.75	0.52	0.76	0.82	0.83	0.30	0.83	**0.84**	0.83	0.88	0.68
*Elephantorrhiza burkei*	0.43	0.59	0.35	0.58	0.49	0.22	0.56	**0.75**	0.66	0.91	0.81
*Grewia* spec	0.48	0.47	0.50	0.60	0.60	0.39	0.62	**0.63**	0.59	0.69	0.55
*Lannea discolor*	0.39	0.37	0.42	0.45	0.47	0.13	0.44	**0.57**	0.53	0.57	0.46
*Mundulea sericea*	–	–	–	–	–	–	–	–	–	0.59	0.21
*Ozoroa paniculosa*	0.33	0.19	0.24	0.44	0.33	0.02	0.50	**0.51**	0.38	0.62	0.25
*Pseudolachnostylis maprouneifolia*	0.42	0.47	0.42	0.31	0.03	0.05	0.50	**0.57**	0.26	0.32	0.07
*Pterocarpus rotundifolius*	0.49	**0.53**	0.45	0.45	0.49	0.32	0.46	0.50	0.50	0.74	0.34
*Terminalia sericea*	0.19	0.31	**0.49**	0.15	0.24	0.05	0.21	0.23	0.35	0.84	0.68
Bare ground/herbs	0.94	0.95	0.94	**0.96**	**0.96**	0.94	0.95	0.95	0.95	0.95	0.96
Mean F1-score	0.50	0.50	0.50	0.56	0.54	0.28	0.57	**0.61**	0.57	0.68	0.47
Cohen’s kappa (κ)	0.67	0.65	0.67	0.71	**0.72**	0.50	0.71	**0.72**	**0.72**	0.79	0.66
Overall accuracy	0.82	0.81	0.82	0.84	0.84	0.73	0.84	**0.85**	**0.85**	0.88	0.82

Best values for each class or category are printed in bold. The outer right two columns display values at 512 × 512 pixel tile size for test images recorded adjacent to the training areas (a) at approximately the same time as the training data set and (b) during the 2022 rainy season.

**Figure 2:**
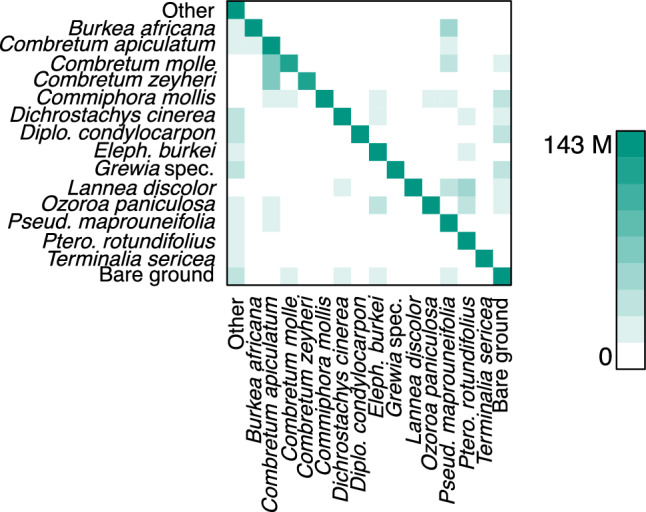
Visualising the confusion matrix for predictions of DeepLabv3 + trained with 512 × 512 pixel input tiles. Predictions were made on three separate 1-ha test plots spatially distant from the training areas. Model output is depicted on the horizontal axis, and true labels on the vertical axis. Colour intensity corresponds to the number of pixels for the respective combination.

**Figure 3:**
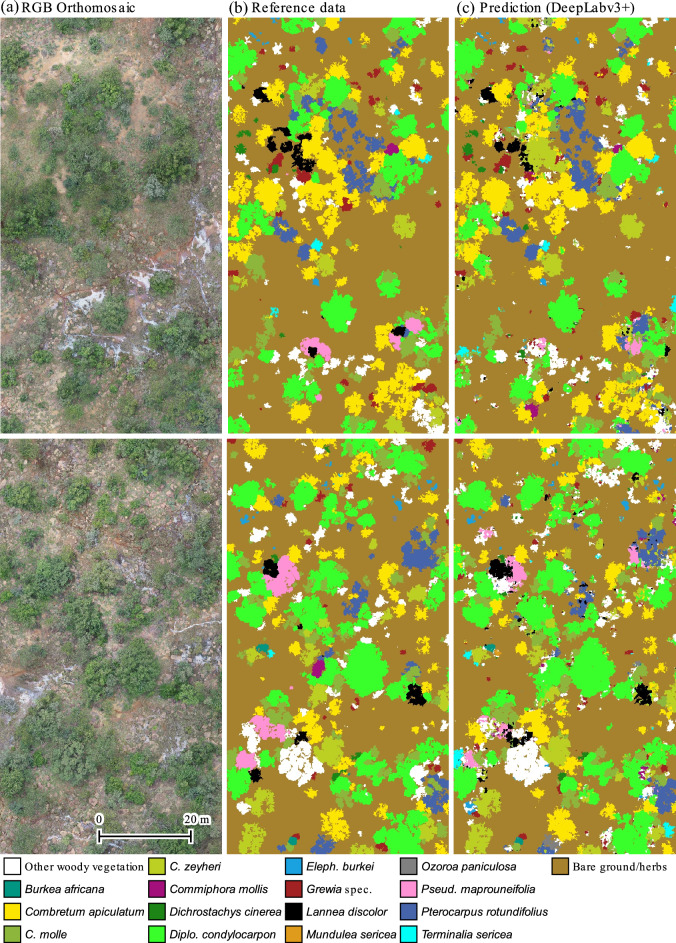
Example prediction of DeepLabv3 + for a ca. 1 ha test plot split in two rows. The CNN was trained with a data set comprising 6 ha of training tiles from blocks 1, 2, and 3. The depicted test orthomosaic was recorded in block 7. The images were split vertically to fit the page format. The upper and lower rows display the left and right half of the plot, respectively.

Best F1-Scores with a mean of 0.61 on the test data were achieved by DeepLabv3 + with the 512 × 512 pixel tile size (Table 2). Individual F1-Scores of this model showed a broad range from 0.23 *(Terminalia sericea)* up to 0.85 *(Burkea africana)* and 0.95 for the background class. It also produced good predictions of *Diplorhynchus condylocarpon* (F1 = 0.84) and *Combretum apiculatum* (F1 = 0.82), the latter of which was most often confused for other *Combretum* species (Fig. 2). The remaining classes were predicted with F1-Scores ranging between 0.50 *(Combretum zeyheri, Pterocarpus rotundifolius)* and 0.75 *(Elephantorrhiza burkei)*. An exemplary prediction of this model on test data is shown in Fig. 3.

The mean patch area and compactness of the patches had no substantial impact on F1-Scores for the respective class (Table 3). Four out of nine models showed a significant linear relationship between F1-Score and total class area within the training data (all *p-*values < 0.05, Appendix E2). Relationships between F1-Score and the class area within the test data were not significant.

**Table 3:** Correlation between mean patch area in ha, total class cover in ha, and compactness of patches (mean smallest circumscribing circle) and F1-score.

Model	Training data			Test data		
	Mean patch area	Class area	Compactness	Mean patch area	Class area	Compactness
U256	186	0.253*	0.874	145	− 0.122	− 0.194
U512	139	0.125	− 0.373	− 36.2	− 0.0615	0.283
U1024	147	0.254*	1.49	127	0.0418	0.957
F256	78.6	0.244	1.13	150	− 0.185	− 0.734
F512	20.3	0.297*	2.57	133	− 0.122	− 0.344
F1024	− 142	0.186	2.94	− 82.4	− 0.0717	0.815
D256	99.3	0.241	1.03	155	− 0.154	− 0.368
D512	115	0.187	− 0.502	115	− 0.174	− 0.743
D1024	5.47	0.248*	1.3	88.1	− 0.081	− 0.073

Values represent the slope of a linear regression line. Asterisks indicate p-values below 0.05. No p-value was below 0.01. Degrees of freedom = 14.

DeepLabv3 + predictions on orthomosaics that were spatially adjacent and temporally close to the training areas achieved the highest F1-Scores: ranging between 0.24 for unspecified woody vegetation, and 0.95 for bare ground/herbaceous vegetation (Table 2). Concordantly, this model reached the highest mean F1-Score (0.68) and best overall accuracy (0.88). Apart from woody background vegetation, only *Combretum zeyheri* and *Pseudolachnostylis maprouneifolia* were predicted with F1-Scores below 0.5. The model achieved good results for *Burkea africana*, *Combretum apiculatum*, *Dichrostachys cinerea*, *Diplorhynchus condylocarpon*, and *Terminalia sericea*. F1-Score for *Elephantorrhiza burkei* was very good (0.91). On the other hand, the model performed poorly on orthomosaics of the same test areas recorded nine months later. Here, predictions reached a mean F1-Score of 0.47 only. With the exception of bare ground/herbs (F1 = 0.96), F1-Scores were consistently lower on the 2022 test data than on test data recorded at the same time as training data.

## Discussion

To our knowledge, this is the first study to assess the suitability of CNNs for semantic segmentation of multiple savanna tree species from UAV RGB imagery. Our results illustrate the suitability of CNN-based tree mapping in savanna ecosystems, albeit within certain restrictions. With clumped sampling and spatially distant test sites, DeepLabv3 + with 512 × 512-pixel input tiles achieved acceptable performance, indicating some potential to deploy trained CNNs at new locations with similar species composition. F1-Scores were similar to those found in other studies segmenting multiple classes^14,39,40^. The choice of CNN architecture had no major impact on model prediction, indicating that properties of the data set rather than model complexity limited model accuracy. Similarity amongst tree species, heterogeneity within tree species, and quality of the orthomosaics were likely the main limiting factors.

Within strict spatial and temporal constraints, DeepLabv3 + has shown the ability to accurately predict individual tree species cover. It achieved a similar F1-Score as a U-Net trained on 51 ha of temperate forest^14^. A major limitation, however, was the poor transferability of the model over time. A low mean F1-Score on orthomosaics recorded during a different month suggests that phenotypic changes during a phenological phase can cause major deterioration of model predictive quality (but see Egli and Höpke^41^). A possible explanation for this deterioration is the high number of species in a savanna ecosystem, which increases the probability of feature overlap. Additional factors affecting model transferability in time could be caused by variations in orthomosaic quality or in light conditions during recording^7^. However, orthomosaics for each block were recorded over several hours or days and, hence, covered some variability in wind, cloud cover, and position of the sun. Consequently, differences between the 2021 and 2022 data set were mainly morphological. For example, plants adjust leaf orientation based on external factors such as moisture and solar irradiation, which change during the vegetation period and are subject to interannual variation^42^. The resulting difference in model prediction accuracy indicates that to monitor species composition across years, training data sets ideally cover a range of phenotypical and phenological variation.

Considerable positive relationships between class training area and class F1-Score may result from the positive impact of training sample size. Large, randomly sampled training data sets increase the likelihood that the heterogeneity of traits within a class is well covered^7^. However, the correlation was not consistent across models and tile sizes. The good results achieved on test data sampled in close temporal and spatial proximity to the training data indicate that semantic segmentation of tree species within an area is possible with reasonable accuracy. This is even the case with a CNN trained on a comparably small training data set if the training data represents the entire area. Consequently, an adequate selection of training locations has an even more substantial impact on the quality of the classification than the mere size of the available data set. Indeed, spatial autocorrelation can cause an overestimation of model performance of up to 28% in temperate forests^43^. While this is to be avoided during model evaluation, maximising similarities between training data and the data that is to be predicted could help improve results when deploying the CNN. We expected large, compact trees to provide additional larger-scale patterns in contrast to smaller, often irregularly growing shrubs. However, mean patch area and patch compactness were unrelated to CNN performance, indicating that other factors determined class-specific prediction accuracy. For example, large individuals or large homogeneous patches may not fit into single image tiles, limiting the maximum usable patch area. Moreover, the overall shape of tree crowns could be less relevant than fine-grained structures. This is in line with a lack of a consistent impact of tile size on mean F1-Scores: While the poor performance of FC-DenseNet with 1024 × 1024-pixel tiles was likely due to the small batch size this configuration allowed, the model performance of U-Net and DeepLabv3 + was not affected by tile size. This is despite the larger tiles more frequently containing entire trees and, hence, providing context information such as the shape of crown outlines. A possible explanation is the lack of distinct large-scale crown structures. Differences between species-specific F1-Scores are likely affected by consistency and distinctness of leaf shape, size, and arrangement^7^. Such could explain the high F1-Score for species such as *Burkea africana* despite its relatively low cover within the training data (appendix B). These factors can also explain the confusion amongst *Combretum* species, as well as between *Burkea africana* and *Pseudolachnostylis maprouneifolia*. In these cases, confusion was somewhat higher between species with leaves or leaflets of similar size (Fig. 2). Confusion with the class “Other” is most likely due to the heterogeneity of such features within this particular class. In the absence of distinct large-scale features, it is crucial to reveal class-specific fine-scale patterns, raising the importance of very high-resolution UAV imagery for the identification of savanna trees to genus or species level.

**Figure 4:**
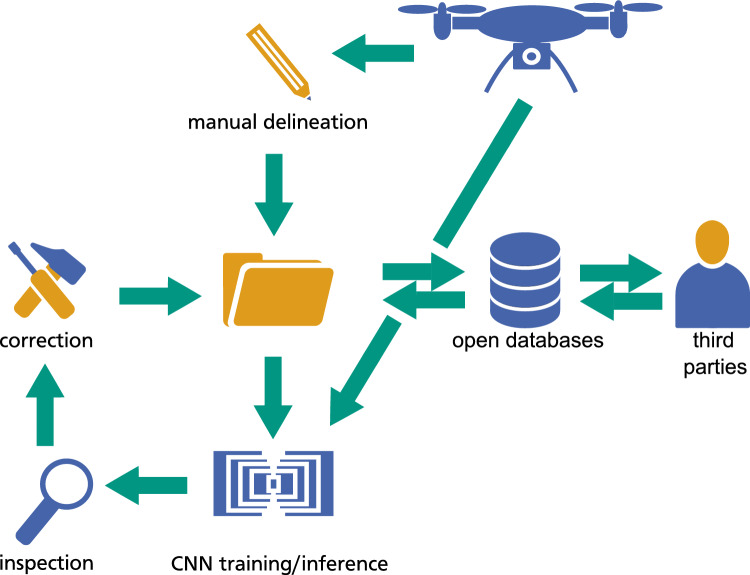
Suggestion for a semi-automatic workflow to generate large data sets for CNN training.

CNN trained exclusively on data recorded during a narrow range of time lacked the robustness required for vegetation monitoring. Still, there is enormous potential to employ CNN to scale up manual tree crown delineation across sites with similar tree species composition and phenological conditions. Also, since class boundaries in savanna ecosystems are predominantly between trees and bare soil, an accurate prediction of bare soil or herb cover can be used to facilitate tree crown delineation. To this end, a CNN can be trained on a small data set. Subsequently, new data can be classified using the trained model. After manual correction, these new data can be used for further training and improvement of the CNN (Fig. 4). In addition to ad hoc delineated images, data sets can be shared to make CNNs more robust and to widen the range of applications^7^. For example, researchers are working on creating diverse databases for CNN training, which will provide means of pre-training, transfer learning, and enable training of CNNs deployable across larger spatial extents and temporal ranges^19,44^.

In this study, sample size and ground sampling distance were restricted by the availability of time and human resources, as is often the case in practice. Sample size, however, is a major factor determining the generalisation capabilities of CNN^7,45^. To make accurate predictions under new conditions, the abundance of heterogeneous samples in the data set plays a key role^46^. Ideally, to increase the robustness of the predictions, CNNs used in vegetation monitoring are trained on data sets recorded at different times and, hence, cover a range of phenotypical and phenological variability^15,47^.

Recent studies suggest that lower ground sampling distance enhances model performance^14,41^. Since the width of leaves/leaflets is usually within a range of millimetres to a few centimetres, sub-centimetre sampling distance at the canopy level will likely greatly improve prediction accuracy. This could be achieved by lowering the UAV flight altitude or by employing a camera with a higher resolution. Despite a relatively low flight altitude, our sampling distance was much coarser compared to studies in temperate forests^14,41^, because most savanna trees are only a few meters in height. Moreover, orthomosaics often contain data gaps due to the complex surface of the vegetation. An increase in side overlap of the flight grid would enrich the dense cloud for the generation of orthomosaics and thereby reduce artefacts and data gaps resulting from undersampling^48^. However, such adjustments of flight parameters would dramatically increase flight time. This may be a considerable disadvantage, especially in cases where flight time exceeds battery capacity, causing interruptions and increased resource requirements. Another solution to reduce the impact of data gaps in orthomosaics could be predicting species on raw UAV images and creating orthomosaics from the resulting species maps. The applicability of such an approach should be tested in further studies. Normalised digital surface models can be calculated from UAV imagery and could be provided as an additional input band for model training and inference^49,50^. Recent studies, however, found the potential improvement of predictions to be minor and potentially outweighed by increased computational complexity^9,14^. In contrast, fusing images recorded across multiple seasons can increase model accuracy significantly^40^. If economically feasible, changes in tree phenology should thus be harnessed to improve classification quality. Finally, the potential impact of JPEG compression loss on orthomosaic quality and, subsequently, on model performance could be assessed in further studies^51^.

## Conclusion

We showed the possibilities and limitations of consumer-grade UAV imagery for semantic segmentation of savanna tree species. Good results can be achieved when the training data set is similar to the data on which predictions are made. Thus, some a priori knowledge of the new data is required. Training data should be recorded during the same time of year and, ideally, on comparable plant communities. Prediction accuracy varies considerably between tree species. Species with distinct leaf size and arrangement, as well as species with high abundance in the training data, are most likely to be predicted with high accuracy. To train models that are robust across space and time, large data sets are required. Orthomosaics need to be of high resolution and high quality to achieve accurate predictions. Currently, model training is computationally intense. While DeepLabV3 + achieved the best results, U-Net can be an option with somewhat lower requirements.

## Methods

### Study area

**Figure 5:**
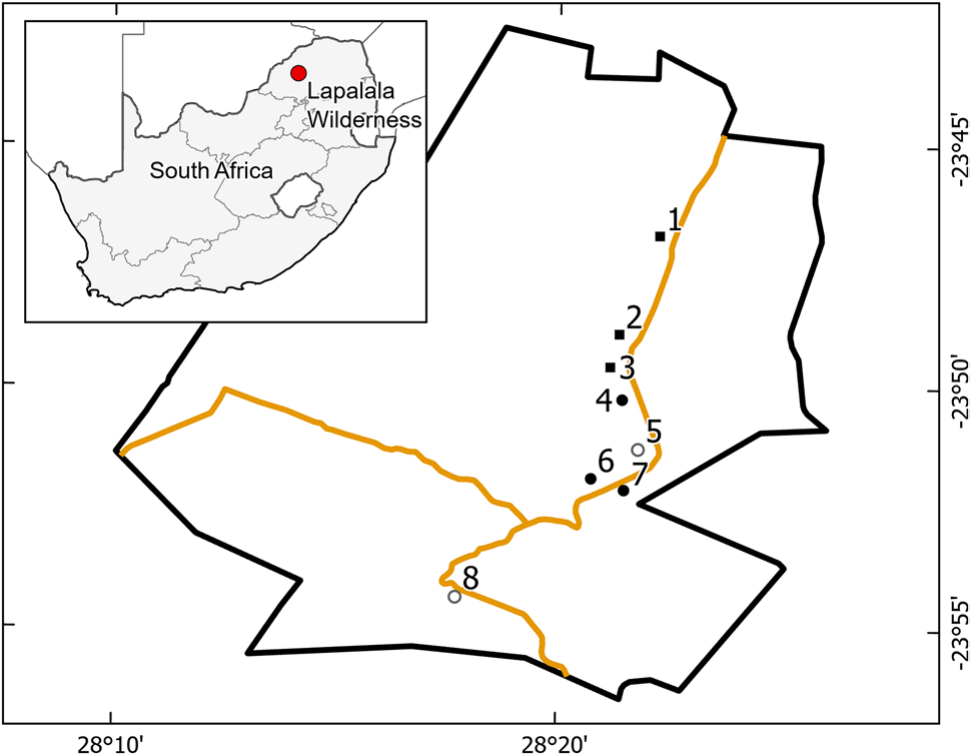
Map indicating the position of the research blocks (N = 8). Symbols indicate (Filled square) full block (6 ha) training area; (Open circle) single plot (1 ha) validation area; and (Filled circle) single plot test area. The green line traces the borders of Lapalala Wilderness, whereas the yellow line indicates the main roads. The inset shows the location of the study area within South Africa. Projection: UTM zone 35S.

Data were acquired in Lapalala Wilderness—a 48′000 ha nature reserve within the Waterberg Biosphere, Limpopo Province, South Africa (Fig. 5). For long-term monitoring of vegetation as part of the Lapalala Elephant Landscape Experiment (LELE) project, eight blocks of 6 ha each were installed along the main road of the reserve. Blocks were divided into 1-ha plots. For this study, we used imagery of the three northernmost blocks as well as one random plot from each of the remaining blocks (Fig. 5).

The region had a subtropical climate, with more than 95% of the 594.4 mm of annual precipitation falling between October and April (Appendix A). Average daily minimum and maximum air temperatures ranged from 18.1 °C to 31.5 °C in February and 2.3 °C to 23.0 °C in July, the warmest and coldest months, respectively. Geology in the reserve was dominated by sedimentary rocks, with local intrusions of basic norite/epidiorite^52,53^. Plant community composition within the reserve was heterogeneous and appeared to be determined mainly by the pH and clay content of the soil^37^. Therefore, locations with soil characteristics as similar as possible were selected for placement of the research blocks. Soils in the blocks were mainly Lithic and Nudilithic Leptosols that developed on red sandstone and diamictite with a sandy matrix (Appendix C)^54^. An exception was the area surrounding block 4, where soils were thicker and could be classified as Acrisols, according to ISRIC World Soil Information and FAO^55^.

The vegetation type covering most of Lapalala Wilderness was described as Waterberg Mountain Bushveld^38^. Woody vegetation in the reserve comprised species such as *Combretum* spp., *Diplorhynchus condylocarpon*, *Pterocarpus rotundifolius*, and *Terminalia sericea*. Overall, more than 60 tree species were found within the research plots (Appendix B).

Woody vegetation in Lapalala Wilderness was affected by browsers such as black rhinoceros *(Diceros bicornis)*, various ungulate species, and recently reintroduced African elephant *(Loxodonta africana)*. Because of poor soils with low pH, grasses in the area had a low nutritional value^37,38^. Grasses lignified at the beginning of the rainy season and, thus, intermediate feeders also browsed seasonally^56,57^. Management practices affecting vegetation included prescribed burning and mechanical bush clearing (Appendix D)^58^.

### Image data collection

Aerial images were recorded in March 2021 and January/February 2022 using a DJI Phantom 4 Pro quadcopter (DJI Sciences and Technologies Ltd., Shenzhen, China) and an integrated RGB camera with a 24 mm lens. The exact date of each flight is listed in Appendix G. With each individual flight, a single 1-ha research plot plus some peripheral areas were mapped. The UAV followed a crisscross pattern at speeds of about 3 m s^−1^, recording with a -65° gimbal angle. The flight route was configured to achieve 95% front overlap and 65% side overlap, as required for the subsequent creation of high-quality orthomosaics^48^. During all flights, an altitude of 40 m was maintained, resulting in a ground sampling distance of about 1.2 cm. Images were saved with JPEG compression.

Orthomosaics were created using Agisoft Metashape v 1.5.4 (Agisoft LLC, St. Petersburg, Russia). To improve the spatial accuracy of the orthomosaics, five reference points were marked at the corners and centre of each plot. Reference point locations were determined using a ppm 10xx GNSS sensor (Pforzheimer Präzisions Mechanik GmbH & Co. KG, Ispringen, Germany) mounted on a tablet, achieving a horizontal accuracy of < 1 m.

### Reference data generation

Woody plants within the plots were mapped in the field in November 2021. Orthomosaics were used as background maps for orientation during tree mapping with ArcGIS Field Maps (ESRI, Redlands, USA). For each individual tree or homogeneous cluster of trees of a single species, we recorded the centre coordinates, taxon name, and approximate crown diameter. Common tree species were identified following Van Wyk and Van Wyk^59^. Species endemic to the region were identified using Coates-Palgrave^60^. Taxon names were checked and updated following the World Flora Online^61^. A comprehensive species list can be found in Appendix B.

**Figure 6:**
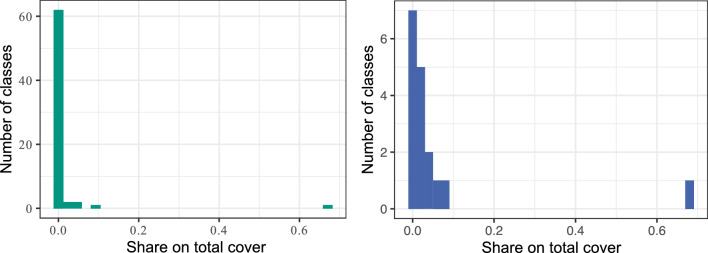
Histograms showing class share by area before and after merging classes. The left panel shows all classes separate; the right panel shows small classes agglomerated. The largest class is bare ground.

Segmentation classes were manually delineated in ArcGIS Pro v 2.9.3 (ESRI, Redlands, USA) based on field data and using the orthomosaics as background. The number of tree species in the plots was high, and their respective abundances were imbalanced, with many covering only tiny fractions of the area (Fig. 6). To reduce the number of small classes, all five species of the genus *Grewia* were merged into one class. Furthermore, species with a share of less than 1% of the total tree cover were combined into a single background class.

Our data set comprised blocks 1, 2, and 3, as well as one random plot from each of the remaining five blocks (Fig. 5). Spatial separation of training areas from validation and test plots was required to minimise spatial autocorrelation which otherwise would have caused significant overestimation of model performance^10,43^. In the first step, this was secured through physical distances of more than 1 km between neighbouring blocks. Here, blocks 1, 2, and 3 were used for training, while three of the remaining single plots were used as testing data sets and two for validation (Fig. 5).

In the second step, one plot each from blocks 1, 2, and 3 was put aside for testing, while all of the single plots 4, 5, 6, 7, and 8 were used for validation. Additional orthomosaics of these test plots were recorded in January 2022 and used as a second test data set. These data sets had no spatial overlap with the training data but were spatially adjacent to training areas and, hence, were expected to have similar characteristics^10^. Test plots from the first field campaign were recorded under similar tree phenological conditions, causing further similarities between training and test data^10,43^.

To train CNNs such as U-Net, Ronneberger et al.^17^ favoured large input tiles over a large batch size. Schiefer et al.^14^, on the other hand, achieved the best results with smaller tiles. To find the best model, we tested multiple tile sizes with side lengths of 256, 512, and 1024 pixels. Non-overlapping square tiles were generated using the GDAL/OGR Python API of OSGeo v 3.2.3. The respective number of tiles per 1-ha plot were 1259, 308, and 74. These were average values since the actual dimensions of the plots showed slight variation.

To improve model generalisation, training data were augmented on the fly. Data augmentation comprised random horizontal flip, rotation by an angle within ± 0.35 rad, random scaling by factors ∊ [0.9, 1.1], random brightness changes by up to $\pm$ 25%, random contrast changes by 50–200%, and random saturation changes within 60–175%. These values were selected after visually inspecting the effect of the augmentation operations and searching for the limits of what we perceived as natural. A preliminary test run showed that wider ranges for these parameters had no benefit on validation accuracy.

### CNN training

For this study, we trained three different CNN architectures: (1) U-Net, (2) FC-DenseNet, and (3) DeepLabv3 + . Details can be found in Appendix F.

CNNs were trained on the bwUniCluster 2.0 cluster computer on Red Hat Enterprise Linux 8.3.1–5 using 40 2.1 GHz CPUs (Intel Xeon Gold 6230) and four CUDA-compatible GPUs (NVIDIA Tesla V100, 8 GB RAM each). These resources allowed for batch sizes of 4, 1, and 4 tiles with 1024-pixel side lengths for U-Net, FC-DenseNet, and DeepLabv3 + , respectively. For training with smaller tiles, batch sizes were increased inversely proportional to tile area. To mitigate the impact of extreme class imbalances, weighted categorical cross-entropy was used as loss function. The weight $w$ for each class $i$ was calculated according to Eq. (1):

1 $${w}_{i}=\frac{1}{{p}_{i}^{x}},$$

where $p$ was the proportional share of class $i$ on the training data set, and $x$ was a parameter that could be optimised. We chose this function since it provided flexibility in relative scaling of class weights while retaining simplicity. Optimalisation of individual class weights is computationally intensive and beyond the scope of this study. Multiple test runs over 80 epochs, with $x$ between 0.001 and 10, showed the fastest growing learning curves on the validation set around 0.1, 0.005, and 0.2, for U-Net, FC-DenseNet, and DeepLabv3 + , respectively. These values for $x$ were used in all further training runs of the models. As optimiser, we selected Adam, which is computationally efficient and suitable for a wide range of problems^62^. After short test runs, including the values 0.1, 10^–2^, 10^–3^, and 10^–4^, the initial learning rate (α) was set to 10^–4^. Default values (β_1_ = 0.9 and β_2_ = 0.999) were used for the remaining parameters. The metric tracked during learning was mIoU, which is a standard metric for segmentation purposes^63^. It is calculated as:

2 $${\text{mIoU}}=\frac{1}{n}\sum_{i=1}^{n}\frac{{O}_{i}}{{U}_{i}},$$

where $n$ is the number of classes, ${O}_{i}$ is the overlap (intersection) between the predicted area for class $i$ and the ground-truth area for the same class (true positives). ${U}_{i}$ is the area of union for these areas, i.e., the sum of true positives, false positives, and false negatives. The number of epochs was limited to a maximum of 300, or 40 consecutive epochs, without improvement of validation mIoU. Models were saved at the end of each epoch. Best models were selected based on maximum validation mIoU after smoothing small-scale fluctuations of the learning curve through local polynomial regression.

Code was written in Python v 3.8.6 using modules TensorFlow with TensorFlow-GPU v 2.8.0^64^, TensorFlow-addons v 0.15.0, and NumPy v 1.22.2.

### Accuracy assessment

To evaluate model performance, predictions of the trained models were compared with manually delineated ground truth data. Model predictions were written to 16 layers with x- and y-offset by 25%, 50%, and 75% of the tile size in the respective dimension. For each test plot, a final prediction was derived through a majority vote. Subsequently, a confusion matrix was calculated based on final predictions and ground truth data. From this confusion matrix, precision (P), recall (R), and F1-Score for each class, as well as overall accuracy, were derived. F1-Score was calculated according to Eq. (3).

3 $${\text{F1}}=2\times \frac{P\times R}{P+R}.$$

Models were operationalised on a machine with GeForce GTX 970 (4 GB) GPU, Intel i7-4790K CPU (4 GHz, 4 cores), and 32 GB RAM.

### Statistical analyses

We used multiple paired t-tests to test for the impact of CNN architecture and input tile dimensions on model performance. Normal distribution of differences was usually met (Appendix E). Furthermore, we calculated the total area covered, mean patch area, and mean smallest circumscribing circle (a measure for compactness of patches) for each class in the ground truth data used for training. We then tested for correlations between these class metrics and corresponding F1 scores. Analyses were carried out in R v 4.2.1 using packages landscapemetrics v 1.5.2 and rstatix v 0.6.0^65–67^.

### Supplementary Information

Supplementary Information.

## Data Availability

All data generated or analysed during this study are included in this published article and its supplementary information files.

## Code availability

All code used during model training and statistical analyses is accessible at: https://github.com/ManuelPopp/LELENet.
