# Supplementary material for: Consumer-grade UAV imagery facilitates semantic segmentation of species-rich savanna tree layers
Source: Sci Rep. 2023 Aug 24;13:13892. doi: 10.1038/s41598-023-40989-7 (PMC10449814; doi:10.1038/s41598-023-40989-7)
Supplement: Supplementary file 1 — Supplementary Information. [file 41598_2023_40989_MOESM1_ESM.pdf]

Consumer-grade UAV imagery facilitates semantic segmentation of species-rich savanna tree layers

Manuel R Popp[1] and Jesse M Kalwij[1,2,3]

[1] *Institute of Geography and Geoecology, Karlsruhe Institute of Technology, Reinhard-Baumeister-Platz 1, 76131 Karlsruhe, Germany*
[2] *Centre for Ecological Genomics & Wildlife Conservation, Department of Zoology, University of Johannesburg, Auckland Park, South Africa*
[3] *Van Hall Larenstein University of Applied Sciences, Velp, Netherlands*

List of figures

A 1 Climate diagram . . . ii
B 1 Class fractions and patch metrics . . . iv
B 2 Example tiles . . . vii
C 1 Soil map . . . ix
C 2 Sedimentary rocks . . . x
D 1 Prescribed burnings . . . xi

List of tables

B 1 Species list . . . iii
B 2 Taxonomy changes . . . iv
E 1 Paired t-tests between model F1-Scores . . . xii
E 2 Wilcoxon signed-rank tests between model F1-Scores . . . xiii
E 3 Linear correlations between F1-Score and class properties . . . xiv
G 4 List of orthomosaics . . . xvi

Contents

A Regional climate **ii**

B Tree species **iii**

C Soil types within the study area **ix**

D Prescribed burnings **xi**

E Supplementary statistical tables **xii**

F Notes on CNN architectures **xv**

G Orthomosaic download locations **xvi**

A Regional climate

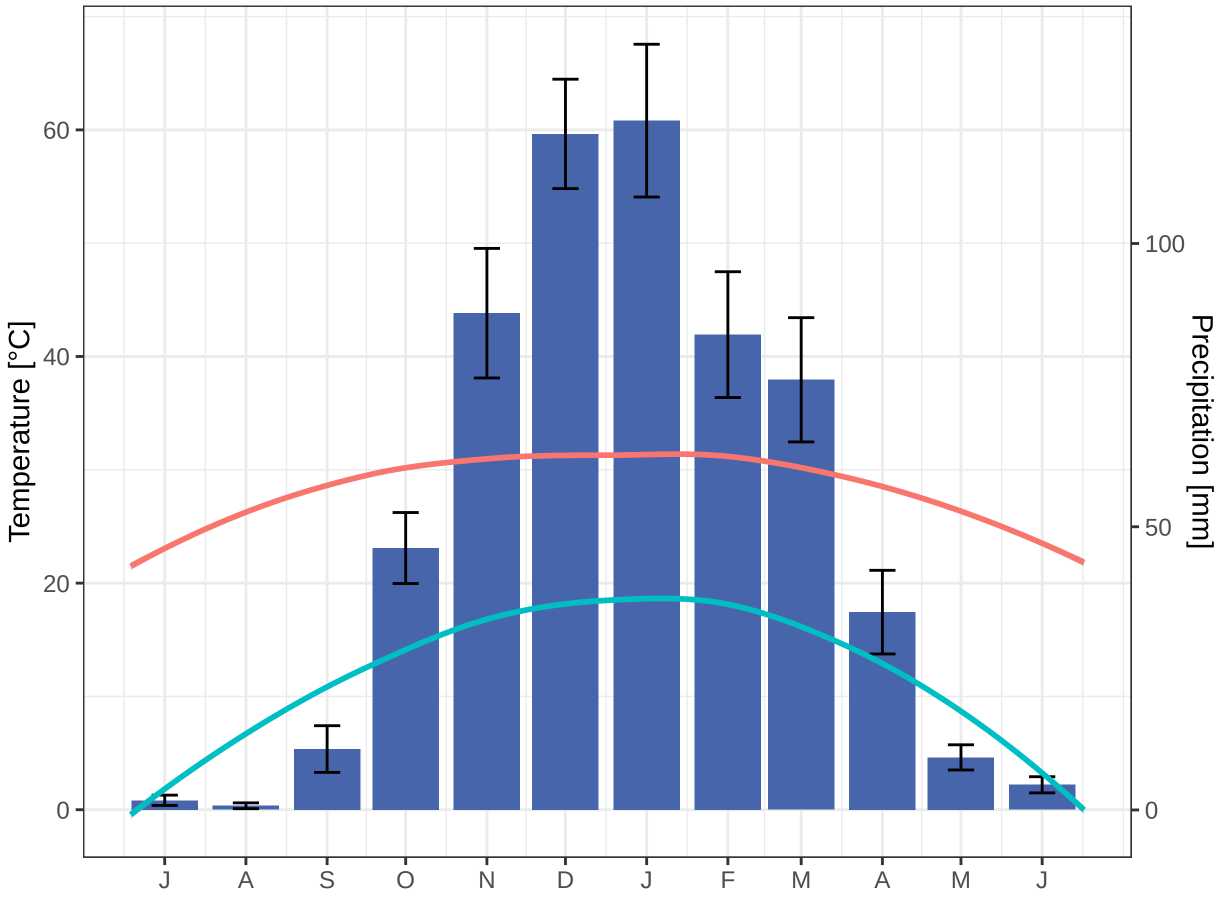

Figure A 1: Regional climate. Lines display daily — T_{min} and — T_{max} measured at Marken, about 20 km north of the study area (Sep 1993–Feb 2022; source: South African Weather Service, 2022). Bars with error bars display monthly precipitation (mean ± SE) measured at Frishgewaagd, a neighbouring property to Lapalala Nature Reserve (July 1990–July 2020; source: Hermann Müller, Lapalala Wilderness, personal communication). Mean annual precipitation amounts to 594 mm.

Figure A 1 shows a climate diagram for the northern Waterberg region. Daily minimum and maximum temperatures were provided by the South African Weather Service. The measurements cover the period from September 1993 to February 2022. The curves display the mean values calculated by day of year. Precipitation was measured at Frishgewaagd, a neighbouring property to Lapalala Nature reserve. A 30-year period from July 1990 to June 2020 was used to calculate average monthly rainfall.

B Tree species

Table B 1: List of all tree species found in the research plots. Taxon names and information were semiautomatically matched and manuelly checked following the World Flora Online Kindt, 2020; WFO, 2022.

Family	Genus	Epithet	Authority	Source
Rubiaceae	*Afrocanthium*	*mundianum*	(Cham. & Schltdl.) Lantz	Bot. J. Linn. Soc. 146: 278 (2004)
Apocynaceae	*Ancylobothrys*	*capensis*	(Oliv.) Pichon	Mém. Inst. Franç. Afrique Noire 35: 297 (1953)
Rhamnaceae	*Berchemia*	*zeyheri*	(Sond.) Grubov	Trudy Bot. Inst. Akad. Nauk S.S.S.R., Ser. 1, Fl. Sist. Vyssh. Rast. 8: 374 (1949)
Capparaceae	*Boscia*	*albitrunca*	(Burch.) Gilg & Benedict	Bot. Jahrb. Syst. 53: 212 1915
Asteraceae	*Brachylaena*	*discolor* subsp. *rotundata*	(S.Moore) Beentje	Kew Bull. 55(1): 27 (2000)
Phyllanthaceae	*Bridelia*	*mollis*	Hutch.	Bull. Misc. Inform. Kew 1912: 100 (1912)
Fabaceae	*Burkea*	*africana*	Hook.	Hooker's Icon. Pl. 6: tt. 593, 594 (1843)
Apiaceae	*Choritaenia*	*capensis*	Benth.	Hooker's Icon. Pl. 13: 24, t. 1231 1877
Combretaceae	*Combretum*	*apiculatum*	Sond.	Linnaea 23: 45 (1850)
Combretaceae	*Combretum*	*hereroense*	Schinz	Verh. Bot. Vereins Prov. Brandenburg 30: 245 (1888)
Combretaceae	*Combretum*	*molle*	R.Br. ex G.Don	Trans. Linn. Soc. London 15: 431 (1827)
Combretaceae	*Combretum*	*zeyheri*	Sond.	Linnaea 23: 46 (1850)
Burseraceae	*Commiphora*	*mollis*	Engl.	Monogr. Phan. 4: 23 (1883)
Euphorbiaceae	*Croton*	*gratissimus*	Burch.	Trav. S. Africa 2: 263 (1824)
Fabaceae	*Dichrostachys*	*cinerea*	(L.) Wight & Arn.	Prodr. Fl. Ind. Orient. : 271 (1834)
Apocynaceae	*Diplorhynchus*	*condylocarpon*	(Müll.Arg.) Pichon	Bull. Mus. Natl. Hist. Nat. ser. 2, 19(4): 368. (1947)
Malvaceae	*Dombeya*	*rotundifolia*	(Hochst.) Planch.	Fl. Serres Jard. Eur. 6: 225 (1851)
Boraginaceae	*Ehretia*	*rigida*	Druce	Rep. Bot. Soc. Exch. Club Brit. Isles 4: 620 (1916 publ. 1917)
Fabaceae	*Elephantorrhiza*	*burkei*	Benth.	London J. Bot. 5: 81 (1846)
Sapotaceae	*Englerophytum*	*magalismontanum*	(Sonder) T.D.Penn.	Gen. Sapotac. : 252 (1991)
Ebenaceae	*Euclea*	*crispa*	(Thunb.) Gürke	Nat. Pflanzenfam. 4(1): 158 (1891)
Ebenaceae	*Euclea*	*crispa* subsp. *linearis*	(Zeyh. ex Hiern) F.White	Bull. Jard. Bot. Natl. Belg.
Ebenaceae	*Euclea*	*natalensis*	A.DC.	Prodr. 8: 218 (1844)
Rubiaceae	*Gardenia*	*volkensii*	K.Schum.	Bot. Jahrb. Syst. 34: 332 (1904)
Malvaceae	*Grewia*	*damine*	Gaertn.	Fruct. Sem. Pl. 2: 113 (1790)
Malvaceae	*Grewia*	*flava*	DC.	Cat. Pl. Horti Monsp. : 113 (1813)
Malvaceae	*Grewia*	*flavescens*	Juss.	Ann. Mus. Natl. Hist. Nat. 4: 91 (1804)
Malvaceae	*Grewia*	*monticola*	Sond.	Linnaea 23: 20 (1850)
Malvaceae	*Grewia*	*rogersii*	Burtt Davy & Greenway	Man. Pl. Transvaal 1: 41 (1926)
Celastraceae	*Gymnosporia*	*buxifolia*	(L.) Szyszyl.	Polypet. Discifl. Rehmann. 2: 34 (1888)
Celastraceae	*Gymnosporia*	*tenuispina*	(Sond.) Szyszyl.	Polypet. Discifl. Rehmann. 2: 33 (1888)
Myrtaceae	*Heteropyxis*	*natalensis*	Harv.	Thes. Cap. 2: 18 (1863)
Annonaceae	*Hexalobus*	*monopetalus*	Engl. & Diels	Monogr. Afrik. Pflanzen-Fam. 6: 56 (1901)
Kirkiaceae	*Kirkia*	*acuminata*	Oliv.	Fl. Trop. Afr. 1: 311 (1868)
Anacardiaceae	*Lannea*	*discolor*	Engl.	Nat. Pflanzenfam. , Nachtr. 1: 213 (1897)
Sapotaceae	*Mimusops*	*zeyheri*	Sond.	Linnaea 23: 74 (1850)
Fabaceae	*Mundulea*	*sericea*	(Willd.) A.Chev.	Compt. Rend. Hebd. Séances Acad. Sci. 180: 1521 (1925)
Myrothamnaceae	*Myrothamnus*	*flabellifolia*	Welw.	Apont. : 578 (1859)
Ochnaceae	*Ochna*	*pulchra*	Hook.	Hooker's Icon. Pl. 6: t. 588 (1843)
Ochnaceae	*Ochna*	*serrulata*	Walp.	Repert. Bot. Syst. 5: 400 (1846)
Anacardiaceae	*Ozoroa*	*paniculosa*	(Sond.) R.Fern. & A.Fern.	Bol. Soc. Brot. , sér. 2, 38: 167 (1965)
Rubiaceae	*Pavetta*	*zeyheri*	Sond.	Fl. Cap. 3: 21 (1865)
Fabaceae	*Peltophorum*	*africanum*	Sond.	Linnaea 23: 35 (1850)
Phyllanthaceae	*Pseudolachnostylis*	*maprouneifolia*	Pax	Bot. Jahrb. Syst. 28: 20 (1899)
Asteraceae	*Psiadia*	*punctulata*	Vatke	Oesterr. Bot. Z. 27: 196 (1877)
Rutaceae	*Ptaeroxylon*	*obliquum*	(Thunb.) Radlk.	Sitzungsber. Math.-Phys. Cl. Kï£¡nigl. Bayer. Akad. Wiss. München 20: 165 (1890)
Fabaceae	*Pterocarpus*	*rotundifolius*	Druce	Rep. Bot. Soc. Exch. Club Brit. Isles 1916: 642 (1917)
Vitaceae	*Rhoicissus*	*revoilii*	Planch.	Monogr. Phan. 5: 469 (1887)
Anacardiaceae	*Sclerocarya*	*birrea*	Hochst.	Flora 27(Bes. Beil.): 1 (1844)
Anacardiaceae	*Searsia*	*leptodictya*	(Diels) T.S.Yi, A.J.Mill. & J.Wen	Molec. Phylogen. Evol. 33: 864 (2004)
Fabaceae	*Senegalia*	*nigrescens*	(Oliv.) P.J.H.Hurter	Plant-book , ed. 3: 1021 (2008)
Loganiaceae	*Strychnos*	*cocculoides*	Baker	Bull. Misc. Inform. Kew 1895: 98 (1895)
Loganiaceae	*Strychnos*	*madagascariensis*	Poir.	Encycl. 8: 696 (1808)
Loganiaceae	*Strychnos*	*pungens*	Soler.	Bot. Jahrb. Syst. 17: 554 (1893)
Loganiaceae	*Strychnos*	*spinosa*	Lam.	Tabl. Encycl. 2: 38 (1794)
Combretaceae	*Terminalia*	*sericea*	Burch. ex DC.	Prodr. 3: 13 (1828)
Fabaceae	*Vachellia*	*nilotica*	(L.) P.J.H.Hurter & Mabb.	Plant-book , ed. 3: 1021 (2008)
Fabaceae	*Vachellia*	*tortilis*	(Forssk.) Galasso & Banfi	Atti Soc. Ital. Sci. Nat. Mus. Civico Storia Nat. Milano 149: 150 (2008)
Rubiaceae	*Vangueria*	*dryadum*	S.Moore	J. Linn. Soc., Bot. 40: 93 (1911)
Rubiaceae	*Vangueria*	*madagascariensis*	J.F.Gmel.	Syst. Nat. ed. 13bis : 367 (1791)
Rubiaceae	*Vangueria*	*parvifolia*	Sond.	Linnaea 23: 58 (1850)
Rubiaceae	*Vangueria*	*triflora*	(Robyns) Lantz	Pl. Syst. Evol. 253: 181 (2005)
Lamiaceae	*Vitex*	*pooara*	Corbishley	Bull. Misc. Inform. Kew 1920: 333 (1920)
Lamiaceae	*Vitex*	*rehmannii*	Gürke	Bull. Herb. Boissier 4: 818 (1896)
Olacaceae	*Ximenia*	*americana*	L.	Sp. Pl. : 1193 (1753)
Olacaceae	*Ximenia*	*caffra*	Sond.	Linnaea 23: 21 (1850)
Rhamnaceae	*Ziziphus*	*mucronata*	Willd.	Enum. Pl. : 251 (1809)

Table B 2: Accepted taxon names and synonyms found in the field guides of van Wyk and van Wyk and Coates Palgrave following the World Flora Online Coates Palgrave et al., 2002; Van Wyk and van Wyk, 2013; WFO, 2022.

Synonym	Source field guide	Accepted name
Euclea linearis Zeyh. ex Hiern	Van Wyk & van Wyk	*Euclea crispa* subsp. *linearis* (Zeyh. ex Hiern) F.White
Grewia bicolor Roth	Van Wyk & van Wyk	*Grewia damine* DC.
Pachystigma triflorum Robyns	Coates Palgrave	*Vangueria triflora* (Robyns) Lantz.

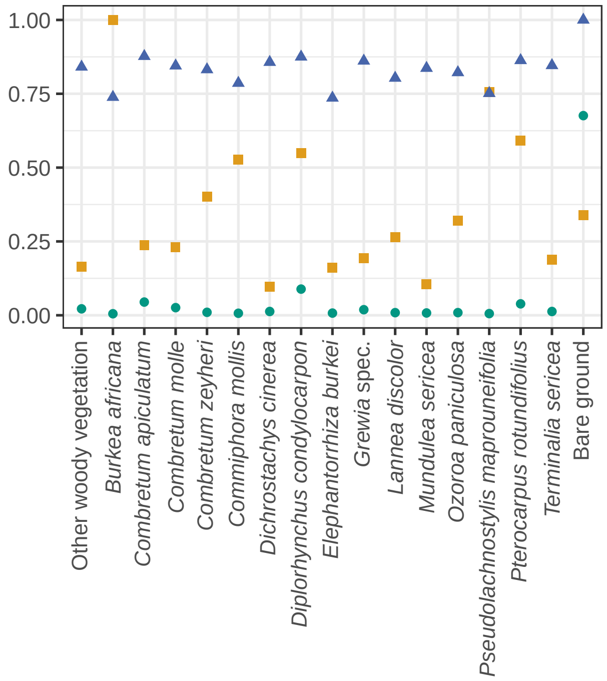

Figure B 1: Class fractions and patch metrics for each class within the training data. ● fraction cover, ▲ mean smallest circumscribing circle, and ■ mean patch area. The latter two were scaled through division by their respective maximum value.

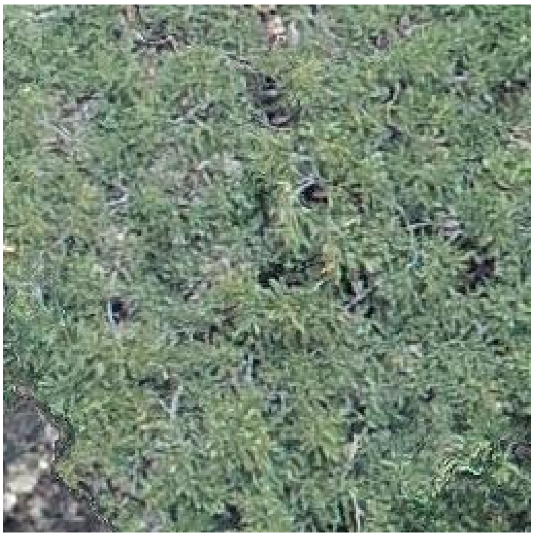

(a) *Burkea africana*

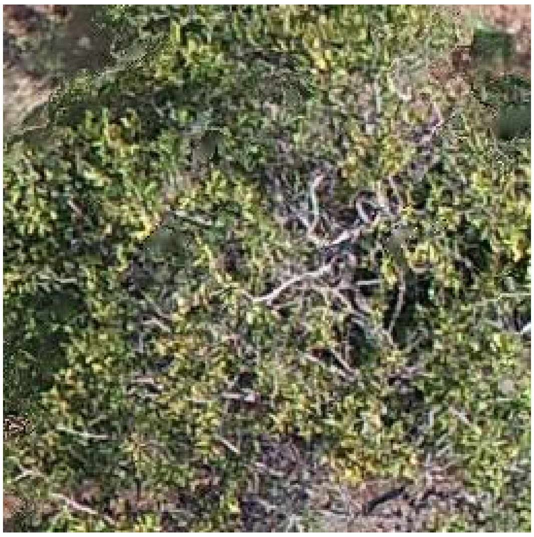

(b) *Combretum apiculatum*

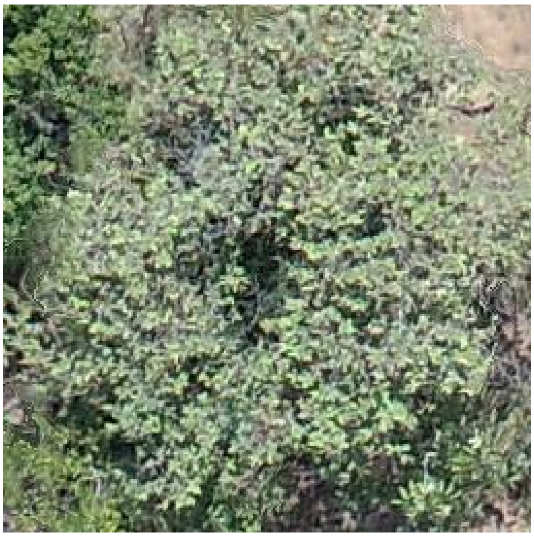

(c) *Combretum molle*

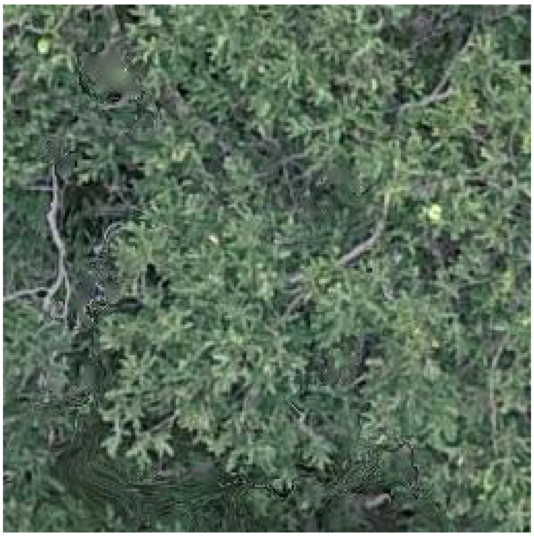

(d) *Combretum zeyheri*

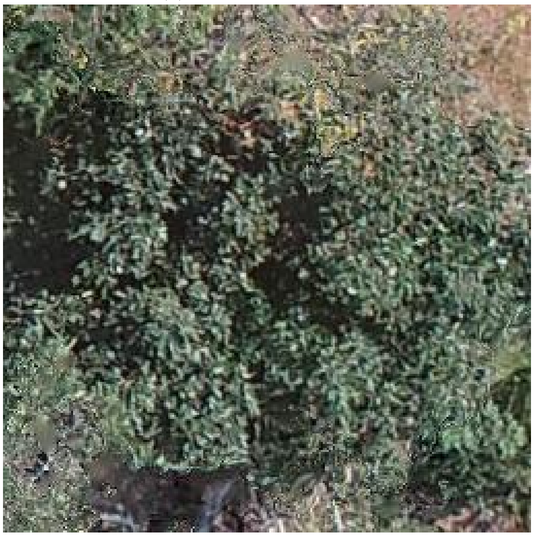

(e) *Commiphora mollis*

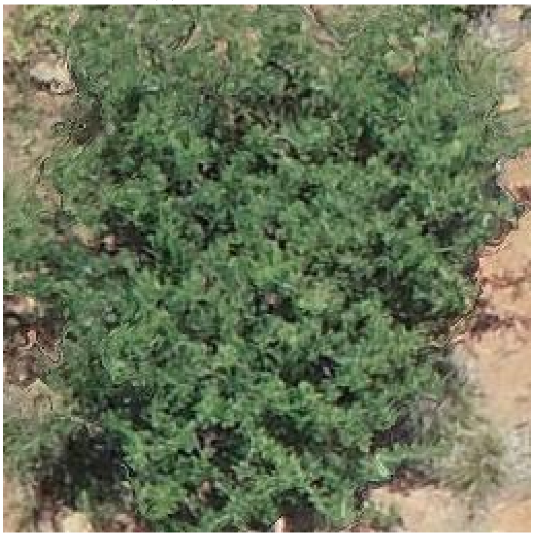

(f) *Dichrostachys cinerea*

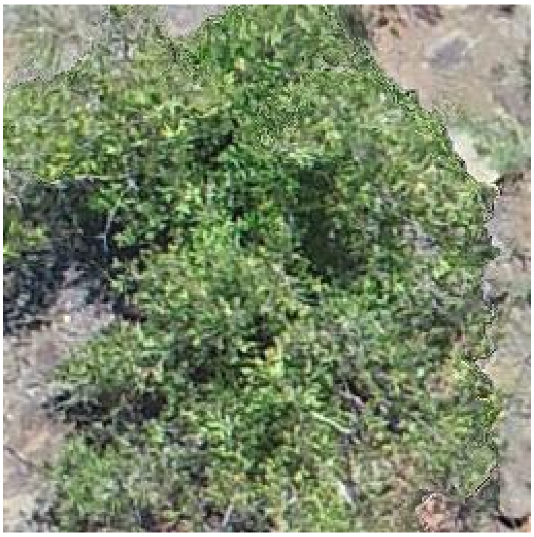

(g) *Diplorhynchus condylocarpon*

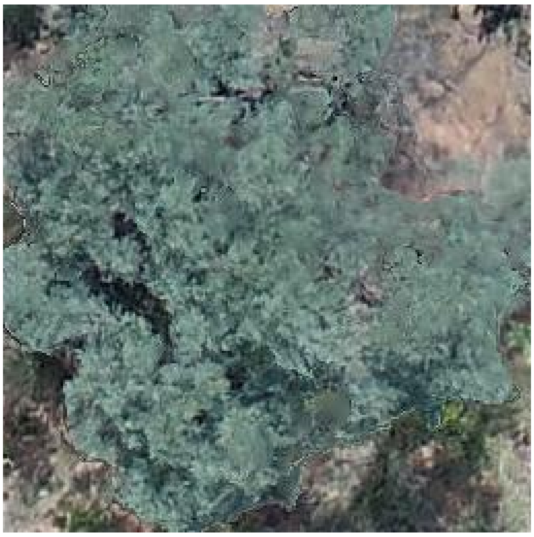

(h) *Elephantorrhiza burkei*

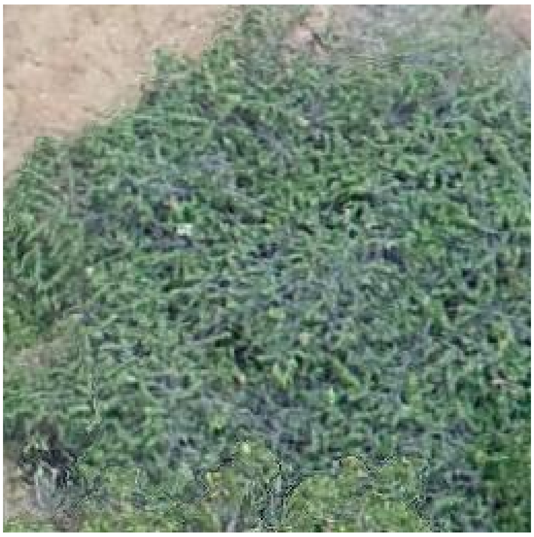

(i) *Grewia* spec.

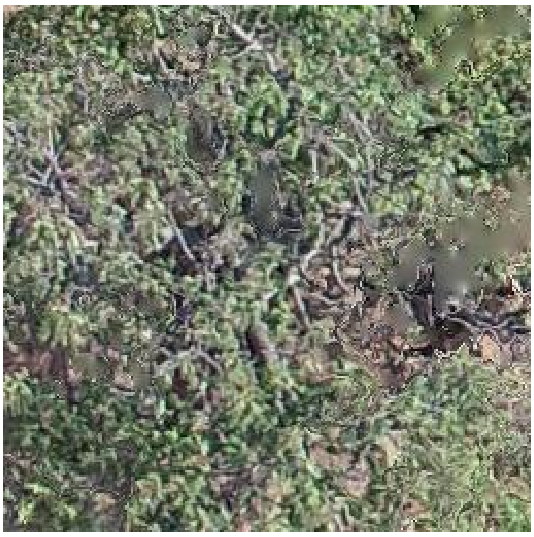

(j) *Lannea discolor*

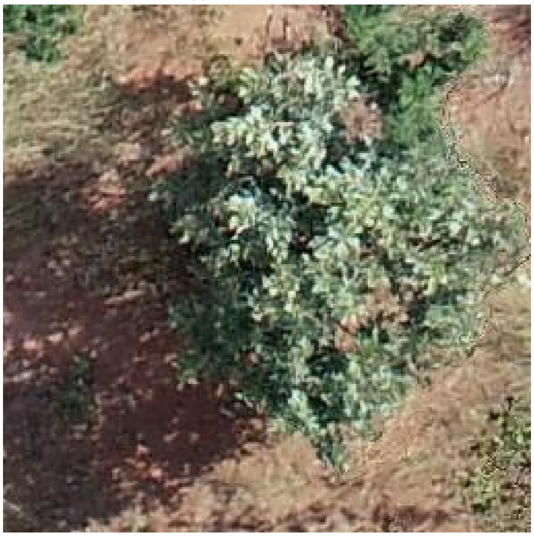

(k) *Mundulea sericea*

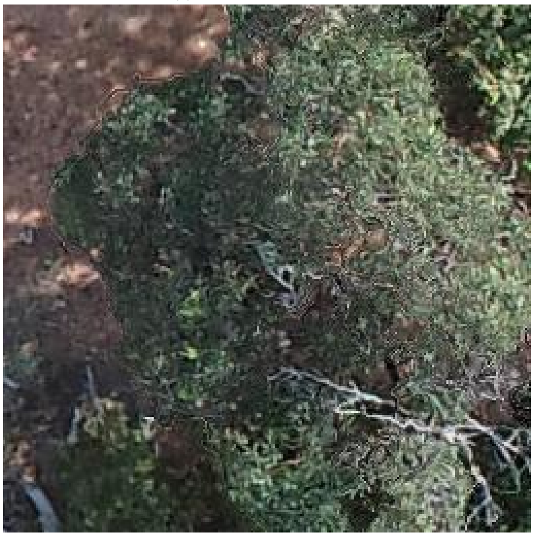

(l) *Ozoroa paniculosa*

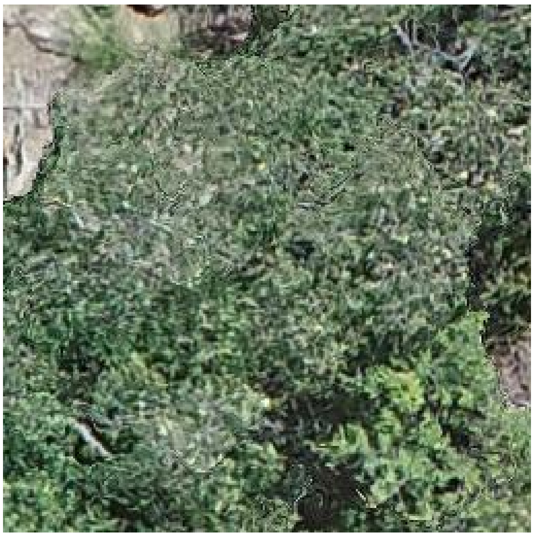

(m) *Pseudolachnostylis maprouneifolia*

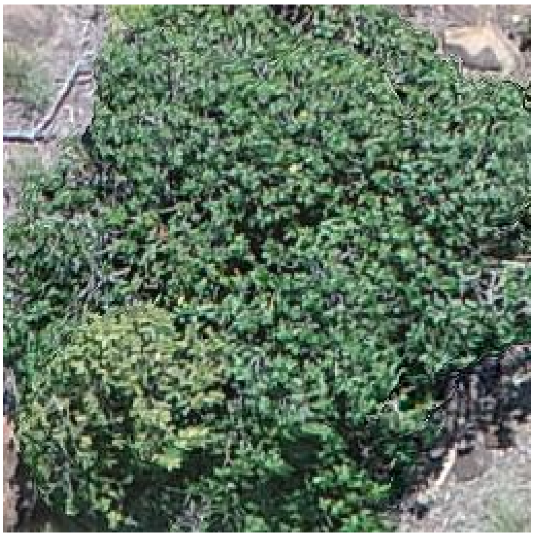

(n) *Pterocarpus rotundifolius*

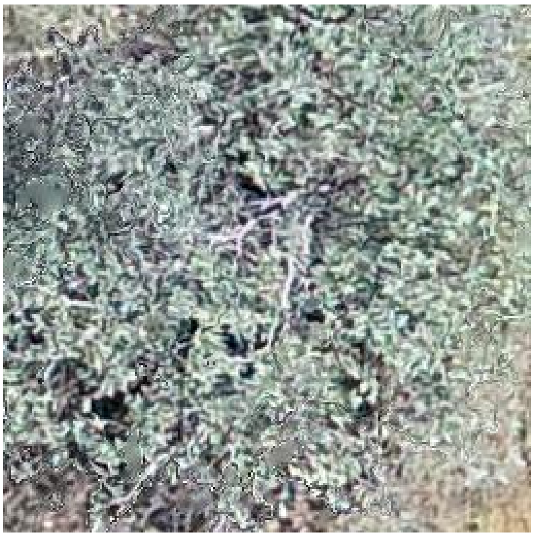

(o) *Terminalia sericea*

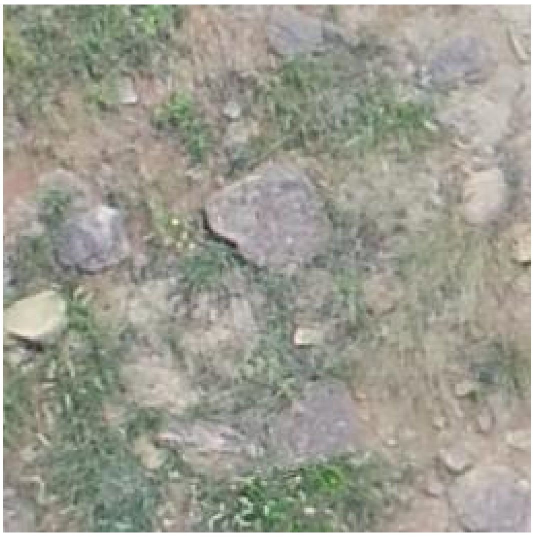

(p) Bare ground

Figure B 2: Manually selected tiles of 256×256 pixel centred on an exemplary individual for each class (except “Other”).

References

Coates Palgrave, Meg et al. (2002). *Palgrave's trees of Southern Africa*. 3rd edition. Century City: imp. 4 Penguin Random House Struik (Pty) Ltd. 1221 pp.

Kindt, Roeland (2020). "WorldFlora: An R package for exact and fuzzy matching of plant names against the World Flora Online taxonomic backbone data". In: *Appl. Plant Sci.* 8.9, e11388. DOI: 10.1002/aps3.11388.

Van Wyk, Braam and Piet van Wyk (2013). *Field guide to trees of Southern Africa*. Cape Town, South Africa: Struik Publishers. 732 pp.

WFO (2022). *World Flora Online*. URL: http://www.worldfloraonline.org (visited on 02/06/2022).

C Soil types within the study area

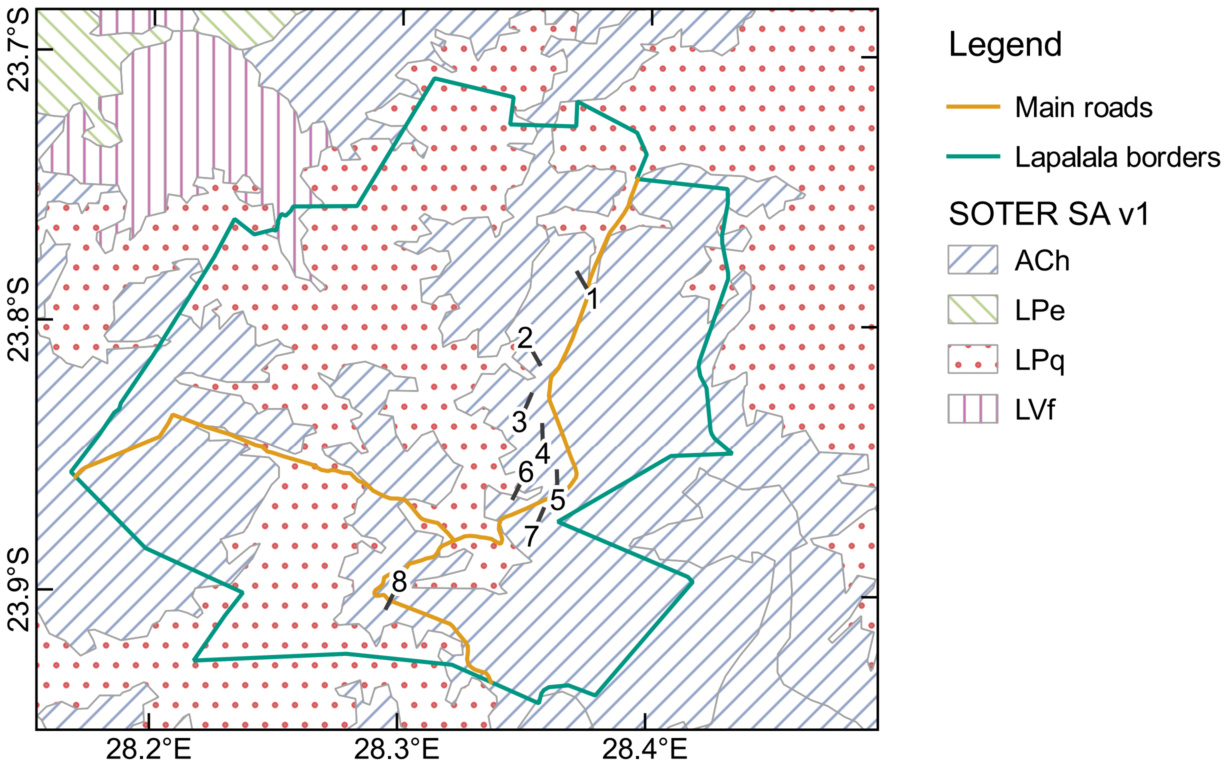

Figure C 1: Map of soil types within the study area. Classification follows the FAO Soil Map of the World FAO, 1988. Soils in the area are Haplic Acrisols (ACh), Eutric Leptosols (LPe), Lithic Leptosols (LPq), and Ferric Luvisols (LVf). Data source: ISRIC ISRIC, 2013. Projection: UTM zone 35S.

Figure fig. C 1 shows soil types found within the study area. All blocks fall into areas marked as Haplic Acrisols (ACh). However, most blocks are close to the boundary between soil types, and field observations showed soils of these blocks were not Acrisols but Lithic and Nudilithic Leptosols on sandstone and diamictite (fig. C 2).

References

FAO (1988). *FAO/Unesco Soil Map of the World, Revised Legend, with corrections.* Tech. rep. Reprinted as Technical Paper 20, ISRIC, Wageningen, 1994. ISBN 90-6672-057-3. Rome: FAO.

ISRIC (2013). *SOTER data model v1.0.* ISRIC–World Soil Information, Wageningen. DOI: 10.17027/ISRIC-WDCSOILS.20180002.

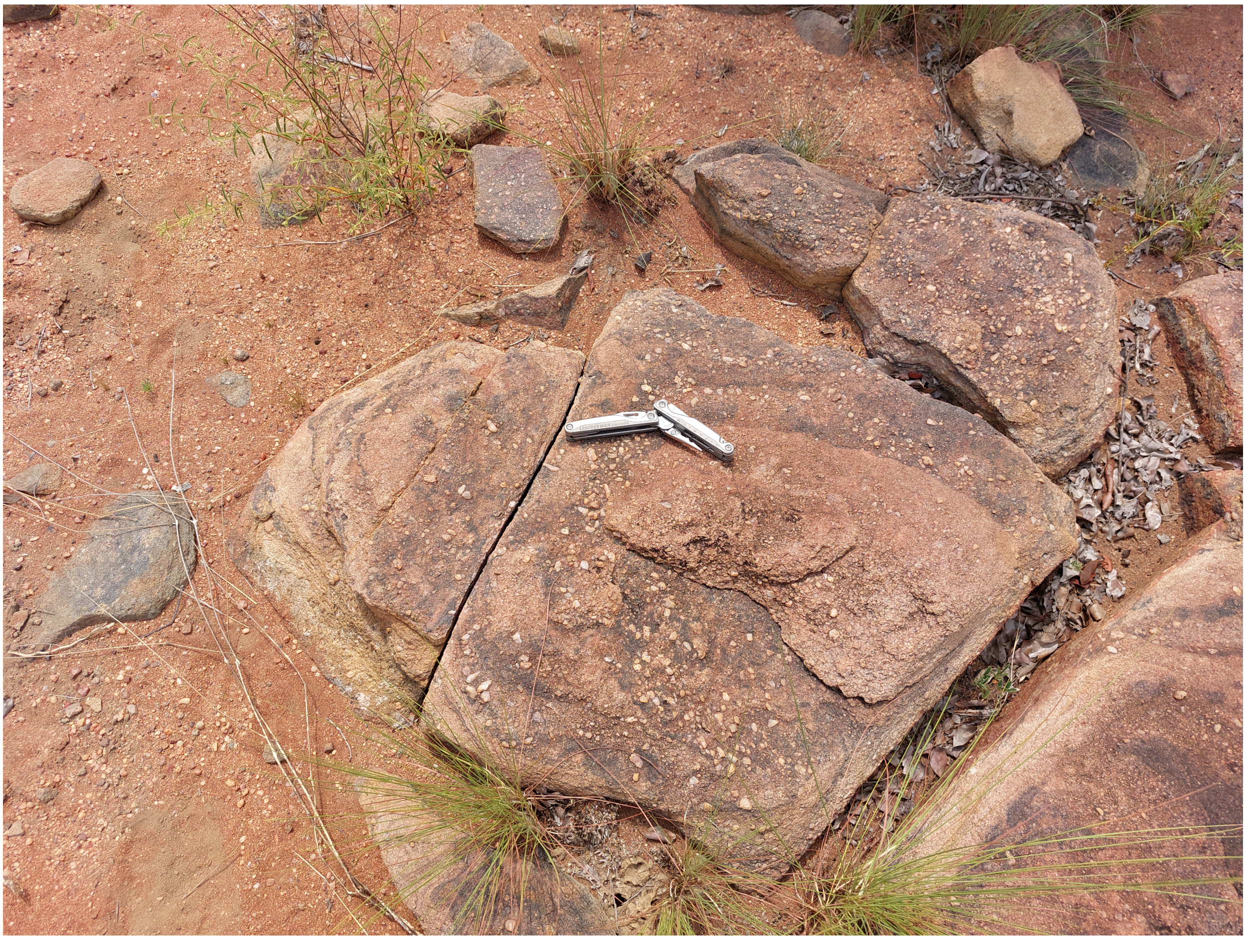

Figure C 2: Sedimentary rocks are the main parent material for soils within the reserve. In some research plots, outcropping rock constitutes a considerable fraction of bare ground. Photo: M. Popp

D Prescribed burnings

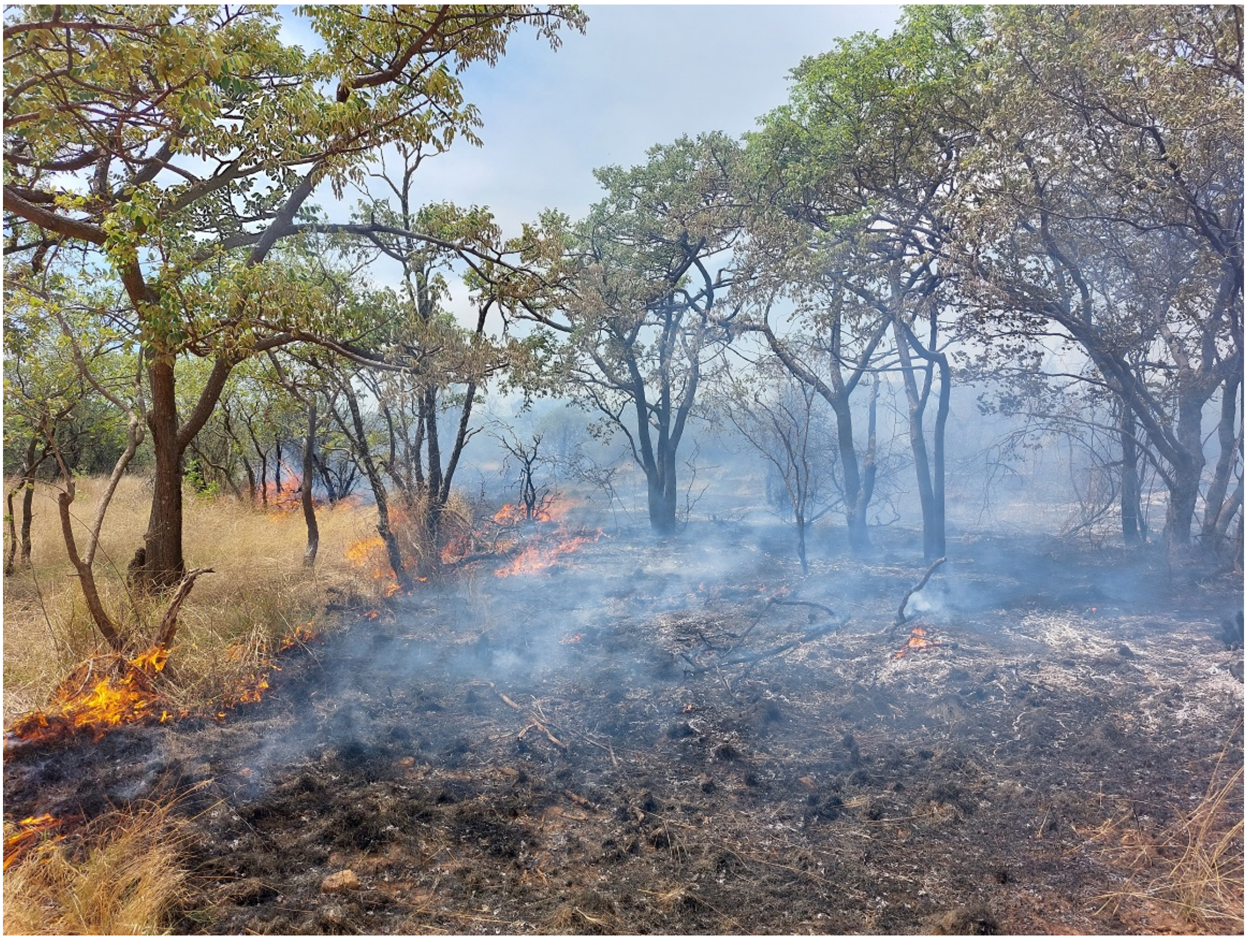

(a) Prescribed burning within the reserve

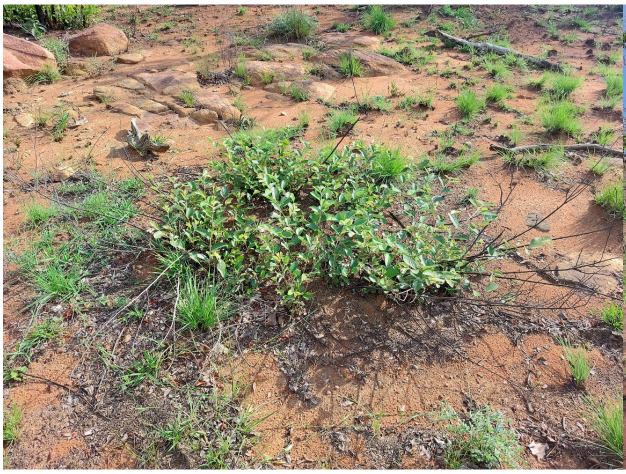

(b) *Grewia rogersii* resprouting after a fire

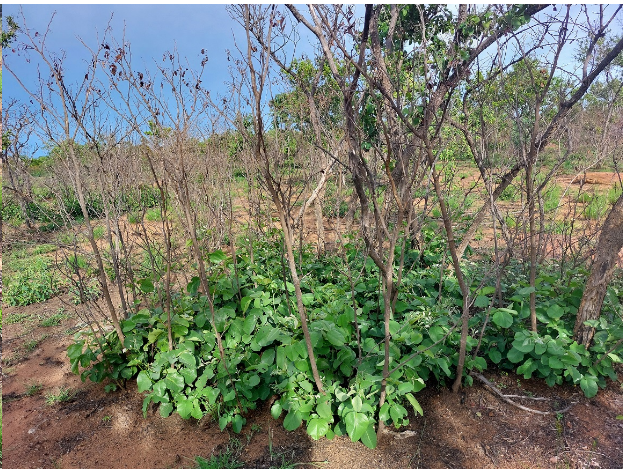

(c) *Pterocarpus rotundifolius* resprouting after a fire

Figure D 1: Prescribed burnings as management practice and two species resprouting after a fire. Individuals resprouting after burning were often observed to grow larger leaves/leaflets than plants of the same species not recently affected by fire. Photos: M. Popp

E Supplementary statistical tables

The following table E 1 shows comparisons between F1-Scores of the models for different image tile sizes. Since test assumptions could not always be met, Wilcoxon signed-rank tests were performed on the same data (table E 2).

Table E 1: a) t-values and b) p-values from paired t-tests between F1-Scores of the models. Tile size is the side length of the image tiles. Gray values indicate comparisons for which the differences were not normally distributed according to a Shapiro-Wilk test ($\alpha = 0.05$). Degrees of freedom = 18.

(a) t-values

CNN		U-Net			FC-DenseNet			DeepLabv3+	
	Tile size	256	512	1024	256	512	1024	256	512
U-Net	512	−0.459							
	1024	−0.19	0.225						
FC-DenseNet	256	3.46	2.32	2.02					
	512	1.48	1.36	1.31	−0.924				
	1024	−6.21	−6.21	−5.8	−7.78	−6.97			
DeepLabv3+	256	4.58	2.86	2.51	0.521	0.856	7.74		
	512	5.58	4.67	3.57	2.68	1.96	8.05	2.66	
	1024	3.29	3.06	2.79	0.678	1.52	8.67	0.136	−1.81

(b) p-values

CNN		U-Net			FC-DenseNet			DeepLabv3+	
	Tile size	256	512	1024	256	512	1024	256	512
U-Net	512	0.65							
	1024	0.85	0.82						
FC-DenseNet	256	0.003	0.03	0.06					
	512	0.16	0.19	0.21	0.37				
	1024	<0.001	<0.001	<0.001	<0.001	<0.001			
DeepLabv3+	256	<0.001	0.01	0.02	0.61	0.40	<0.001		
	512	<0.001	<0.001	0.002	0.02	0.07	<0.001	0.02	
	1024	0.004	0.007	0.01	0.51	0.14	<0.001	0.89	0.09

Table E 2: a) Test statistic and b) p-values from paired Wilcoxon signed-rank tests between F1-Scores of the models. Tile size is the side length of the image tiles. Degrees of freedom = 18.

(a) Test statistic

CNN		U-Net			FC-DenseNet			DeepLabv3+	
	Tile size	256	512	1024	256	512	1024	256	512
U-Net	512	72							
	1024	55	96						
FC-DenseNet	256	166	146	158					
	512	171	149	153	98				
	1024	2	2	4	0	2			
DeepLabv3+	256	180	159	166	118	95	190		
	512	190	180	173	158	148	190	170	
	1024	169	168	157	110	128	190	97	39

(b) p-values

CNN		U-Net			FC-DenseNet			DeepLabv3+	
	Tile size	256	512	1024	256	512	1024	256	512
U-Net	512	0.37							
	1024	0.11	0.98						
FC-DenseNet	256	0.003	0.04	0.009					
	512	0.001	0.03	0.02	0.92				
	1024	<0.001	<0.001	<0.001	<0.001	<0.001			
DeepLabv3+	256	<0.001	0.008	0.003	0.37	1	<0.001		
	512	<0.001	<0.001	<0.001	0.009	0.03	<0.001	0.001	
	1024	0.002	0.002	0.01	0.57	0.2	<0.001	0.95	0.02

Table E 3: Linear correlations between F1-Score and class properties as expressed through the metrics mean patch area (in ha), total class area (in ha) and compactness of patches (mean smallest circumscribing circle for patches of the class).

(a) Training data

Model	Tile size	Mean patch area		Class area		Compactness	
		slope	p-value	slope	p-value	slope	p-value
U-Net	256	186	0.20	0.25	0.04	0.87	0.63
	512	139	0.33	0.12	0.32	−0.37	0.83
	1024	147	0.28	0.25	0.02	1.49	0.36
FC-DenseNet	256	78.6	0.62	0.24	0.06	1.13	0.55
	512	20.3	0.91	0.29	0.04	2.57	0.22
	1024	−142	0.27	0.18	0.09	2.94	0.05
DeepLabv3+	256	99.3	0.52	0.24	0.06	1.03	0.58
	512	115	0.42	0.18	0.12	−0.50	0.77
	1024	5.47	0.97	0.24	0.04	1.30	0.47
DL 2021	512	−12.0	0.93	0.17	0.21	0.82	0.67
DL 2022	512	−25.6	0.89	0.15	0.36	−0.28	0.90

(b) Test data

Model	Tile size	Mean patch area		Class area		Compactness	
		slope	p-value	slope	p-value	slope	p-value
U-Net	256	145	0.25	−0.12	0.24	−0.19	0.85
	512	−36.0	0.77	−0.06	0.55	0.28	0.77
	1024	127	0.27	0.04	0.67	0.95	0.30
FC-DenseNet	256	150	0.25	−0.18	0.08	−0.73	0.49
	512	133	0.38	−0.12	0.33	−0.34	0.78
	1024	−82.4	0.47	−0.07	0.44	0.81	0.36
DeepLabv3+	256	155	0.24	−0.15	0.15	−0.36	0.73
	512	115	0.34	−0.17	0.07	−0.74	0.44
	1024	88.1	0.49	−0.08	0.44	−0.07	0.94
DL 2021	512	79.1	0.56	0.10	0.33	0.13	0.90
DL 2022	512	22.9	0.89	0.13	0.31	0.57	0.66

F Notes on CNN architectures

U-Net consists of an encoder and a symmetric decoder path. The former one comprises multiple layers of 3×3 unpadded convolutions, each followed by a rectified linear unit (ReLU), and a max pooling operation. The number of feature channels is doubled each step of this contracting part. Within the expansive (decoder) path, the feature map is upsampled. Each step is followed by a 2×2 convolution and concatenation with the corresponding feature map of the encoder path that has similar dimensions. These steps are followed by two 3×3 convolutions and a ReLU (Ronneberger et al., 2015). This original structure was modified by introducing batch normalisation before each ReLU of the convolution blocks, similar to Schiefer et al. (2020).

FC-DenseNet consists of multiple dense blocks (DBs) with batch normalisation, ReLU, 3×3 convolution, and a dropout layer. In the downsampling path, each dense block (DB) is followed by a transition down (TD). A TD comprises batch normalisation, ReLU, 1×1 convolution, dropout, and 2×2 max pooling. In the subsequent upsampling path, each DB is preceeded by a transition up (TU). These consist of a single 3×3 transposed convolution. Skip connections are introduced to recover fine-grained information from the downsampling layers during upsampling (Jégou et al., 2017). FC-DenseNet architectures can be built with different numbers of dense layers, filters, and growth rates, which is the factor by which the number of feature maps increases with depth (Jégou et al., 2017). We conducted preliminary test runs using the original FC-DenseNet103 architecture proposed by Jégou et al. (2017) and the shallower FC-DenseNet56, as well as the version used by Torres et al. (2020). FC-DenseNet103 required the longest time per training epoch but achieved the best mean intersection over union (mIoU) on the validation set and, hence, was selected for all further tests. It is built from 103 convolutional layers with 4 to 15 layers for each DB and a growth rate of 16.

DeepLabv3+ also comprises of encoder and decoder (Chen et al., 2018). It is based on DeepLabv3, which uses atrous convolution for feature extraction. Here, the kernel is dilated in order to increase its perceptive field without increasing computational cost, allowing to extract features at an arbitrary resolution (Chen et al., 2017). DeepLabv3+ uses a simple decoder to improve upon the DeepLabv3 structure. A thorough description of the model is given by Chen et al. (2018). In this study, we used a DeepLabv3+ with the ResNet50 backbone, which is considered a state-of-the-art model for semantic segmentation (He et al., 2016; Veras et al., 2022).

References

Chen, Liang-Chieh et al. (2017). “Rethinking Atrous Convolution for Semantic Image Segmentation”. In: *arXiv preprint*. DOI: 10.48550/arXiv.1706.05587.

Chen, Liang-Chieh et al. (2018). “Encoder-decoder with atrous separable convolution for semantic image segmentation”. In: *Proceedings of the European conference on computer vision (ECCV)*, pp. 801–818. DOI: 10.48550/arXiv.1802.02611.

He, Kaiming et al. (2016). “Deep Residual Learning for Image Recognition”. In: 2016 IEEE Conference on Computer Vision and Pattern Recognition (CVPR), pp. 770–778. DOI: 10.1109/CVPR.2016.90.

Jégou, Simon et al. (2017). “The one hundred layers tiramisu: Fully convolutional DenseNets for semantic segmentation”. In: 2017 IEEE Conference on Computer Vision and Pattern Recognition Workshops (CVPRW), pp. 1175–1183. DOI: 10.1109/CVPRW.2017.156.

Ronneberger, Olaf, Philipp Fischer, and Thomas Brox (2015). “U-Net: Convolutional networks for biomedical image segmentation”. In: *Medical Image Computing and Computer-Assisted Intervention – MICCAI 2015*. Ed. by Nassir Navab et al. Lecture Notes in Computer Science. Cham: Springer International Publishing, pp. 234–241. DOI: 10.1007/978-3-319-24574-4_28.

Schiefer, Felix et al. (2020). “Mapping forest tree species in high resolution UAV-based RGB-imagery by means of convolutional neural networks”. In: *ISPRS J. Photogramm. Remote Sens.* 170, pp. 205–215. DOI: 10.1016/j.isprsjprs.2020.10.015.

Torres, Daliana Lobo et al. (2020). “Applying fully convolutional architectures for semantic segmentation of a single tree species in urban environment on high resolution UAV optical imagery”. In: *Sensors* 20.2 (2), p. 563. DOI: 10.3390/s20020563.

Veras, Hudson Franklin Pessoa et al. (2022). “Fusing multi-season UAS images with convolutional neural networks to map tree species in Amazonian forests”. In: *Ecol. Inform.* 71, p. 101815. DOI: 10.1016/j.ecoinf.2022.101815.

G Orthomosaic download locations

Table G 4: List of orthomosaics. All orthomosaics were shared as publicly available layer package on ArcGIS Online using the tag “LELE Project”.

Layer Package Name	Date (date of flight)	AGOL link
ortho_B1_1	17-Mar-21	https://www.arcgis.com/home/item.html?id=1ceb77990f964f26806e37f550e1c9dc
ortho_B1_2	17-Mar-21	https://www.arcgis.com/home/item.html?id=e8da0bb90b84475d94f6e6a80cdf31a8
ortho_B1_3	19-Mar-21	https://www.arcgis.com/home/item.html?id=84e432128dda478d9e57cd4b0f465298
ortho_B1_4	19-Mar-21	https://www.arcgis.com/home/item.html?id=4afa295b176d4baabd85c87d14fd0d97
ortho_B1_5	19-Mar-21	https://www.arcgis.com/home/item.html?id=89c90238989c42418d351811a0f3c273
ortho_B1_6	19-Mar-21	https://www.arcgis.com/home/item.html?id=efddb3a556f7448b8139a63d10d804e7
ortho_B2_1	15-Mar-21	https://www.arcgis.com/home/item.html?id=17573dd3a43749ffa7bcc698e6992d7e
ortho_B2_2	15-Mar-21	https://www.arcgis.com/home/item.html?id=cb4b2c5964fd43c883d0a05b469b66c4
ortho_B2_3	14-Mar-21	https://www.arcgis.com/home/item.html?id=f7c40798c19b4515ab4917cfe5c2b190
ortho_B2_4	15-Mar-21	https://www.arcgis.com/home/item.html?id=829314feb52d4985837598952b5489cb
ortho_B2_5	15-Mar-21	https://www.arcgis.com/home/item.html?id=dccb40e8c647473d9f42c57158214a9c
ortho_B2_6	14-Mar-21	https://www.arcgis.com/home/item.html?id=e2f8984ed43f4cd49f7d87ac5ec38940
ortho_B3_1	17-Mar-21	https://www.arcgis.com/home/item.html?id=b337aff99c794c69af32af15ee5e8049
ortho_B3_2	17-Mar-21	https://www.arcgis.com/home/item.html?id=293241ad737d4d418c81cf7b578e2691
ortho_B3_3	16-Mar-21	https://www.arcgis.com/home/item.html?id=e553d661be43406297b6bad4b619aa00
ortho_B3_4	16-Mar-21	https://www.arcgis.com/home/item.html?id=d26b1b3d0fb94dcea26b3624a8f3ec10
ortho_B3_5	16-Mar-21	https://www.arcgis.com/home/item.html?id=599f5befc4474e0781772d5df1b9be76
ortho_B3_6	16-Mar-21	https://www.arcgis.com/home/item.html?id=bb4863d6dd1b41768588892d47a0acd1
ortho_B4_1	13-Mar-21	https://www.arcgis.com/home/item.html?id=3ecf2b751c67470f9b7bc4e9e9ccfc0c
ortho_B5_5	26-Mar-21	https://www.arcgis.com/home/item.html?id=d5f423d3d9d849079028cccbba1ed112
ortho_B6_2	10-Mar-21	https://www.arcgis.com/home/item.html?id=6a62c769322b4ebea1214894b7b47bab
ortho_B7_5	08-Mar-21	https://www.arcgis.com/home/item.html?id=140810e45ed541a4829e2c5402361a11
ortho_B8_5	06-Mar-21	https://www.arcgis.com/home/item.html?id=a990d8796987466aa78f80e194ba6512
ortho_B1_1_22	31-Jan-22	https://www.arcgis.com/home/item.html?id=a366da0370a64e9aad98b64b0b7e32cf
ortho_B2_3_22	07-Feb-22	https://www.arcgis.com/home/item.html?id=67474612c6ae4760b5d0db65f0031d0e
ortho_B3_2_22	25-Jan-22	https://www.arcgis.com/home/item.html?id=301dad76b58143318b661caa8888b5eb
